# Molecular Age Estimation: Current Perspectives and Future Considerations

**DOI:** 10.3390/ijms27073104

**Published:** 2026-03-29

**Authors:** Muriel Tahtouh Zaatar, Rashed Alghafri, Rima Othman, Amira Ahmed, Mounir Alfahel, Mohammed Alhashimi, Mahmod Alsabagh, Aryaman Dayal, Shamma Kamal, Hiba Khamis, Talal Mansour, Lali Rhayem, Khaled Zeidan

**Affiliations:** 1 Department of Biological and Physical Sciences, American University in Dubai, Dubai P.O. Box 28282, United Arab Emirates; amira.ahmad@mymail.aud.edu (A.A.); mouniralfahel@yahoo.com (M.A.); mohammed.alhashimi15@mymail.aud.edu (M.A.); mahmod.alsabagh@mymail.aud.edu (M.A.); aryaman.dayal@mymail.aud.edu (A.D.); shamma.kamal@mymail.aud.edu (S.K.); hiba.khamis@mymail.aud.edu (H.K.); talal.mansour@mymail.aud.edu (T.M.); lali.rhayem@mymail.aud.edu (L.R.); khaled.zeidan@mymail.aud.edu (K.Z.); 2 Dubai Police International Center for Forensic Sciences, Dubai P.O. Box 1493, United Arab Emirates; r.alghafri@hotmail.co.uk; 3 Department of Pulmonary Medicine and Critical Care, Johns Hopkins University, Baltimore, MD 21205, USA

**Keywords:** age estimation, DNA, RNA, proteomics

## Abstract

Age estimation is an important component of forensic investigation, with applications in criminal casework, immigration assessments, and disaster victim identification. Determining whether an individual is a minor or an adult, or estimating the age at death of unidentified remains, can have significant legal and humanitarian implications. Traditional forensic age estimation methods rely primarily on anthropological and radiological assessment of skeletal development and degeneration; however, these approaches may be limited by subjectivity, population-specific reference standards, and reduced precision in adult age estimation. In recent years, molecular biomarkers have emerged as promising complementary tools for age prediction. Molecular approaches, including DNA methylation profiling, Y-chromosome-associated markers, RNA-based biomarkers, mitochondrial DNA alterations, proteomic signatures, and telomere length analysis, reflect biological processes associated with aging and may provide objective indicators that can be measured from biological samples. Among these methods, DNA methylation-based models currently demonstrate the strongest predictive performance and represent the most extensively studied molecular strategy for forensic age estimation. Nevertheless, several challenges remain before widespread forensic implementation can be achieved, including tissue specificity, environmental influences on biomarker stability, population variability, and the need for robust validation across laboratories and forensic sample types. This review summarises the current molecular approaches investigated for forensic age estimation, evaluates their biological basis and methodological limitations, and discusses their potential integration into forensic workflows. While molecular techniques offer promising avenues for improving age estimation, further standardisation, validation, and careful interpretation are required before they can be routinely applied in forensic practice.

## 1. Introduction

Forensic age estimation is an interdisciplinary field at the intersection of forensic science, molecular biology, anthropology, and computational analysis. Accurate determination of an individual’s age has important legal and humanitarian implications in contexts such as criminal investigations, immigration and asylum assessments, human trafficking cases, and disaster victim identification [1,2,3]. In many forensic scenarios, establishing whether an individual is a minor or an adult can significantly influence legal outcomes and the allocation of protective services. Consequently, the development of reliable, objective, and scientifically validated methods for age estimation remains an important priority within forensic research and practice.

Traditionally, forensic age estimation has relied primarily on anthropological and radiological assessment of skeletal development and degeneration [4,5,6]. These approaches evaluate biological indicators such as dental eruption patterns, epiphyseal fusion, and age-related morphological changes in skeletal structures. Although widely used and historically well established, traditional methods can be influenced by observer subjectivity, population-specific reference standards, and environmental factors including nutrition, disease burden, and socioeconomic conditions [4]. Furthermore, adult age estimation remains particularly challenging because degenerative skeletal changes often occur with substantial inter-individual variability, resulting in relatively broad age intervals that may limit their forensic utility [7,8].

Advances in molecular biology over the past two decades have introduced a paradigm shift toward biomarker-based age estimation. Molecular aging signatures arise from cumulative biological processes including epigenetic modification, genomic instability, transcriptional regulation, mitochondrial mutation accumulation, proteomic alteration, and telomere attrition. These molecular features offer the potential to quantify aging at the cellular level, providing an objective complement to traditional morphological assessments [9,10]. The emergence of high-throughput sequencing technologies and bioinformatic modelling has further accelerated this transition, enabling researchers to identify reproducible molecular patterns associated with chronological and biological aging.

Among molecular approaches, DNA methylation analysis has emerged as one of the most promising strategies, demonstrating relatively high accuracy across multiple tissue types [11]. Parallel developments in RNA profiling, proteomics, and mitochondrial genomics have expanded the range of potential biomarkers, each reflecting distinct biological dimensions of the aging process. Importantly, these techniques may be applicable even when only small or degraded biological samples are available, addressing a major limitation of traditional skeletal-based methods [12,13]. However, despite rapid technological progress, several challenges remain, including variability across populations, tissue-specific effects, methodological standardization, and the need for validation within forensic operational settings.

In addition to molecular innovation, the integration of computational approaches such as machine learning and artificial intelligence is reshaping forensic age estimation. These tools enable the analysis of complex multi-omic datasets, facilitating the development of predictive models that may outperform single-marker strategies [14,15]. As the field continues to evolve, a multidisciplinary perspective that combines molecular biology, statistical modelling, and forensic methodology will be essential to ensure both scientific rigor and legal admissibility.

This review therefore aims to provide a comprehensive and critical synthesis of current molecular approaches to forensic age estimation, evaluation of methodological principles, forensic applicability, and emerging research directions. Specifically, we examine advances in DNA methylation profiling, Y-chromosome-specific markers, RNA-based biomarkers, mitochondrial DNA analysis, proteomic strategies, and telomere length assessment. By integrating current evidence and highlighting areas requiring further validation, this expanded review seeks to clarify the role of molecular age estimation within modern forensic science and to identify future avenues for research and translational implementation.

## 2. Materials and Methods

This narrative review synthesises current molecular approaches used for forensic age estimation and evaluates their relevance for forensic applications. A structured literature search was conducted using PubMed, Scopus, and Web of Science to identify relevant peer-reviewed publications published between January 2000 and March 2025. Search terms included combinations of the keywords relevant to review topic. Additional studies were identified through manual screening of reference lists from relevant articles. Studies were included if they investigated molecular biomarkers associated with chronological age, evaluated predictive modelling approaches for age estimation, or discussed the forensic applicability of such biomarkers, particularly in forensically relevant biological materials such as blood, saliva, semen, bone, teeth, or degraded samples. Studies focusing exclusively on clinical aging without forensic relevance, publications lacking methodological detail, and non–peer-reviewed sources were excluded. For the purposes of this review, forensic age estimation refers to the estimation of an individual’s chronological age or age-at-death from biological evidence in medico-legal contexts, including criminal investigations, immigration or asylum assessments, and disaster victim identification.

## 3. Operational Considerations in Forensic Molecular Age Estimation

In forensic practice, age estimation methods must operate under conditions that differ substantially from controlled laboratory environments. Biological material recovered from crime scenes or medico-legal investigations may be degraded, environmentally exposed, present in trace quantities, or derived from mixed biological sources. Factors such as postmortem interval (PMI), environmental exposure, temperature, humidity, microbial activity, and storage conditions can influence the stability of molecular biomarkers and therefore affect the accuracy of age prediction models [13,16]. In addition, forensic samples frequently contain low-template or inhibited DNA and RNA, which may limit the applicability of certain analytical methods or introduce additional variability in molecular measurements [12,17].

The type and origin of biological material also represent important considerations for molecular age estimation. Forensic evidence may include blood, saliva, semen, bone, teeth, or trace biological material, each with distinct cellular composition and biomarker profiles. Many molecular aging markers exhibit tissue-specific behavior, meaning that prediction models developed using one tissue type may not perform reliably when applied to another [9,18]. Furthermore, forensic samples may contain cell mixtures originating from multiple contributors, complicating interpretation and potentially affecting the accuracy of biomarker-based predictions.

Another critical consideration involves the reporting and interpretation of age estimates in forensic contexts. Unlike research settings where continuous age prediction may be sufficient, forensic applications often require decisions based on specific thresholds, such as determining whether an individual is a minor or an adult [19]. Consequently, age estimation methods must provide not only point predictions but also prediction intervals and measures of uncertainty that reflect both biological variability and methodological error [10]. Robust quality control procedures, including assessment of DNA or RNA integrity and appropriate normalization strategies for molecular assays, are also essential to ensure analytical reliability.

Finally, the translation of molecular age estimation methods into routine forensic practice requires extensive validation and standardization. Methods must demonstrate reproducibility across laboratories, robustness to degraded samples, and consistent performance across diverse populations [17,18]. Considerations related to legal admissibility, transparency of statistical models, and clear communication of uncertainty are equally important to ensure that molecular age estimation results can be interpreted appropriately within medico-legal frameworks [19,20].

## 4. Traditional Methods of Age Estimation

Traditional anthropological and radiological approaches have formed the foundation of forensic age estimation for decades, providing structured frameworks for assessing skeletal development and degeneration [6]. These methods rely on observable morphological changes that occur throughout the human lifespan, allowing forensic practitioners to estimate age ranges based on biological maturation and senescence. Despite significant technological advances, traditional techniques remain widely used due to their accessibility, historical validation, and applicability to skeletal remains recovered from forensic contexts [2,4].

Anthropological age estimation methods differ substantially between sub-adult and adult populations. In sub-adults, age estimation is often based on developmental indicators such as dental eruption patterns, epiphyseal fusion, and long bone growth trajectories, which occur in relatively predictable sequences [2,4]. Dental development has demonstrated strong correlations with chronological age and remains one of the most reliable indicators in juvenile individuals. However, developmental rates may be influenced by nutrition, socioeconomic status, and genetic background, introducing potential bias when reference standards derived from specific populations are applied to individuals from different geographic or ethnic origins [4].

Adult age estimation presents considerably greater challenges due to increased biological variability and the gradual nature of skeletal degeneration. Morphological changes in structures such as the pubic symphysis, auricular surface, sternal rib ends, and cranial sutures are commonly assessed to estimate adult age-at-death [7,8]. Methods such as the Suchey–Brooks pubic symphysis system and rib phase analysis have improved consistency compared with earlier qualitative approaches, yet substantial inter-observer variability persists [7,8]. Degenerative processes are influenced by lifestyle factors including physical activity, occupation, trauma history, and chronic disease, which may accelerate or delay skeletal aging independently of chronological age [4,16]. As a result, adult age estimations often produce broad age intervals that may limit their evidentiary value in forensic casework.

Radiological methods have been developed to address some of the limitations associated with direct morphological assessment, particularly in living individuals. Atlases such as Greulich–Pyle and scoring systems like Tanner–Whitehouse allow practitioners to compare radiographic images with established developmental standards to estimate skeletal age [19]. These techniques are frequently applied in medico-legal contexts, including immigration assessments and age verification cases. However, radiographic approaches are subject to several limitations. Population-specific reference datasets may not accurately reflect global diversity, leading to potential systematic bias when applied across different demographic groups [4]. Furthermore, ethical considerations regarding exposure to ionizing radiation have prompted debate about the routine use of imaging in non-medical forensic assessments, particularly when informed consent and clinical necessity are unclear [21].

Another significant limitation of traditional methods lies in their dependence on relatively intact skeletal structures. Fragmented remains, burned bones, or cases involving minimal biological material may preclude reliable morphological analysis. Additionally, anthropological methods typically require specialized expertise and extensive training, and subjective interpretation can introduce inconsistencies between examiners [20]. Efforts to standardize scoring systems and develop statistical models have improved reproducibility; however, the inherent variability of human skeletal aging continues to pose challenges.

Despite these limitations, traditional approaches remain indispensable within forensic science. Rather than being replaced entirely, they are increasingly viewed as complementary to emerging molecular techniques. Integrating morphological assessment with biomolecular analysis may provide a more comprehensive understanding of biological age, combining macroscopic structural information with molecular indicators of cellular aging. This integrative perspective reflects a broader shift within forensic research toward multidisciplinary methodologies capable of improving accuracy while maintaining scientific transparency and legal defensibility.

## 5. DNA Methylation

DNA methylation has emerged as the most extensively studied molecular biomarker for forensic age estimation. Age-associated changes in DNA methylation occur primarily at CpG dinucleotide sites, where the addition or removal of methyl groups influences gene regulation and chromatin structure. These epigenetic modifications accumulate progressively across the lifespan and exhibit measurable correlations with chronological age [9,21]. Unlike stochastic DNA damage, many methylation changes occur in reproducible patterns across individuals, enabling the development of epigenetic clocks, statistical models that estimate chronological age based on methylation patterns across multiple genomic loci [22].

Age-related methylation changes have been identified in numerous genomic regions, including genes involved in development, cellular differentiation, and metabolic regulation. Commonly reported loci associated with age prediction include CpG sites within genes such as ELOVL2, FHL2, KLF14, TRIM59, and ASPA, which have demonstrated strong correlations with chronological age in multiple studies [9,17]. These markers form the basis of many targeted forensic age prediction panels, which aim to balance predictive accuracy with analytical simplicity.

Several analytical platforms have been employed to measure DNA methylation in forensic age estimation studies. Early discovery studies relied on genome-wide methylation arrays, such as the Illumina HumanMethylation450K and EPIC arrays, which enable high-throughput screening of hundreds of thousands of CpG sites across the genome [22,23]. These platforms are primarily used to identify candidate loci associated with aging.

For forensic applications, however, targeted approaches are more commonly used because they require smaller DNA quantities and are more compatible with degraded samples. Techniques such as pyrosequencing, methylation-specific PCR (MSP), and quantitative PCR-based assays have been widely applied to measure methylation levels at selected CpG loci [1]. More recently, massively parallel sequencing (MPS) technologies have been introduced to improve multiplexing capacity and analytical sensitivity, enabling simultaneous analysis of multiple age-associated markers from limited forensic material [17].

These targeted approaches are particularly relevant for forensic casework, where biological samples may be degraded or present in low quantities. Targeted panels designed for specific forensic sample types have therefore become an important focus of current research.

One of the key challenges in DNA methylation-based age estimation is tissue specificity. Methylation patterns vary across cell types because epigenetic regulation reflects tissue-specific gene expression profiles. As a result, age prediction models developed using one tissue type may not perform reliably when applied to another [9].

To address this issue, some studies have developed multi-tissue epigenetic clocks, the most widely known being the model proposed by Horvath, which demonstrated that methylation patterns across numerous CpG sites could predict chronological age across multiple tissues with a mean absolute error of approximately 3–4 years [23]. While genome-wide clocks provide valuable insights into aging biology, forensic applications generally require simplified targeted marker panels that can be applied to specific forensic sample types.

Several forensic methylation panels have therefore been developed for commonly encountered biological materials such as blood, saliva, semen, and buccal cells, with reported prediction errors typically ranging between 3 and 6 years depending on the marker panel, tissue type, and age range studied [9,18]. These panels often include a small number of highly informative CpG sites selected to maximize predictive accuracy while minimizing analytical complexity.

Despite promising predictive performance, several factors must be considered before DNA methylation-based age estimation can be reliably implemented in forensic practice. One important issue involves environmental and lifestyle influences on epigenetic patterns. Factors such as smoking, alcohol consumption, chronic disease, and environmental exposures have been shown to influence DNA methylation profiles, potentially introducing variability into age prediction models [22,24].

Another challenge relates to sample degradation and DNA quality. Forensic samples may be exposed to environmental stressors that affect DNA integrity, potentially influencing methylation measurements. Although several studies have demonstrated that methylation markers can remain detectable in degraded samples, analytical protocols must incorporate robust quality control measures to minimise technical artefacts [17].

Equally important is the need for external validation across independent populations and laboratories. Many methylation-based age prediction models have been developed using relatively homogeneous cohorts, which may limit their generalisability across different populations. Establishing standardised laboratory protocols, validated marker panels, and transparent statistical modelling approaches will therefore be essential for ensuring reproducibility and legal admissibility in forensic contexts [18].

Overall, DNA methylation currently represents the most mature molecular approach to forensic age estimation. Compared with other molecular biomarkers, methylation-based models have demonstrated relatively low prediction error and have been successfully applied to several forensically relevant biological tissues [9,10]. However, important limitations remain. Tissue-specific effects, environmental influences, population variability, and differences in analytical platforms can all affect predictive performance.

Consequently, while DNA methylation shows strong potential for forensic age estimation, further research is required to improve population calibration, inter-laboratory reproducibility, and assay standardisation. Future developments are likely to focus on refining targeted methylation panels suitable for degraded forensic samples and integrating epigenetic markers with other molecular indicators of aging to enhance predictive robustness.

## 6. Y-Chromosome Markers

Y-chromosomal biomarkers represent a specialised molecular approach to forensic age estimation that is applicable exclusively to male individuals. Because the Y chromosome is inherited paternally and does not undergo recombination across most of its length, age-associated molecular changes occurring within Y-linked loci may accumulate in distinct patterns over time. Recent research has therefore investigated Y-chromosomal CpG methylation (Y-CpG) markers as potential indicators of chronological age in male forensic samples [25,26,27].

Several studies have identified age-associated methylation patterns within Y-linked genes such as NLGN4Y and DDX3Y, where CpG methylation levels show measurable correlations with chronological age [26,27]. Targeted methylation assays combined with statistical modelling have demonstrated that Y-CpG markers can contribute to age prediction in male-derived biological materials such as blood or semen. In some experimental datasets, prediction models based on Y-linked methylation markers have produced mean absolute errors comparable to early autosomal methylation panels [25,27]. However, the number of informative loci available on the Y chromosome is considerably smaller than that available in autosomal genomes, which limits the complexity and predictive power of Y-chromosome-based models.

One potential advantage of Y-chromosomal markers is their male specificity, which may be useful in forensic samples containing DNA mixtures. In cases involving mixed biological material, Y-specific markers may allow the selective analysis of male DNA components, potentially improving the interpretability of age-associated signals derived from male contributors.

In addition to epigenetic markers, structural alterations in the Y chromosome have also been explored as potential indicators of aging. One of the most widely studied phenomena is mosaic loss of the Y chromosome (mLOY), which refers to the age-related loss of the Y chromosome in a subset of somatic cells [28,29,30]. The prevalence of mLOY increases with advancing age and has been observed in peripheral blood cells of aging men.

Although mLOY has attracted interest as a potential biomarker of aging, its interpretation is complicated by several biological and environmental confounders. Studies have demonstrated that the frequency of mLOY is strongly influenced by factors such as smoking, chronic inflammation, environmental exposure, and age-related disease, meaning that observed Y-chromosome loss may reflect broader health-related processes rather than chronological aging alone as assessed by miRNA assays [28,29,30,31]. Consequently, mLOY may introduce systematic bias rather than random variation in age estimation models if these confounding influences are not adequately controlled.

Furthermore, the detection of mLOY typically requires genome-wide sequencing or cytogenetic approaches capable of identifying chromosomal mosaicism, which may limit its practicality for routine forensic analysis. For these reasons, mLOY is currently better regarded as a research biomarker associated with aging biology rather than a validated forensic age estimation method.

Overall, Y-chromosomal markers should be considered complementary tools rather than standalone approaches within molecular age estimation frameworks. Their applicability is restricted to male individuals, and the limited number of informative Y-linked loci constrains the predictive capacity of models based solely on Y-chromosome markers. In addition, environmental exposures and health-related factors influencing Y-chromosomal changes may introduce systematic biases that complicate interpretation.

Compared with autosomal DNA methylation panels, which currently demonstrate the strongest predictive performance for age estimation, Y-chromosomal approaches remain less extensively validated. Their primary value may therefore lie in supplementary analysis of male-specific signals, particularly in forensic samples containing mixed DNA or in combination with autosomal methylation markers.

Future research will likely focus on refining Y-linked methylation panels, improving computational modelling approaches, and evaluating their performance in realistic forensic scenarios. However, at present, Y-chromosomal biomarkers should be regarded as emerging supplementary indicators rather than primary forensic age estimation tools.

## 7. RNA-Based Techniques

Age-related molecular changes are not limited to genomic or epigenetic alterations but are also reflected in transcriptional activity. Several classes of non-coding RNAs, particularly microRNAs (miRNAs), long non-coding RNAs (lncRNAs), and circular RNAs (circRNAs), have been investigated as potential biomarkers for forensic age estimation because they regulate gene expression and participate in cellular aging pathways [32,33,34]. These molecules influence post-transcriptional gene regulation and are involved in processes such as cellular senescence, immune function, and metabolic control, all of which may change with increasing age.

Among these RNA species, miRNAs have received the greatest attention in forensic age estimation research. Numerous studies have reported age-associated expression patterns in specific miRNAs measured in peripheral blood, suggesting that transcriptional signatures may provide measurable indicators of chronological age [12,34]. In addition, lncRNAs have been linked to chromatin remodelling and epigenetic regulation, and some have shown age-related expression changes in human tissues [33,35]. More recently, circRNAs have also been investigated due to their increased stability relative to other RNA species and their potential to persist in degraded biological samples [12,36].

RNA-based age estimation studies typically rely on techniques that quantify RNA expression levels across selected biomarkers. Common analytical platforms include quantitative reverse transcription PCR (RT-qPCR), microarray analysis, and RNA sequencing technologies, which allow detection and quantification of age-associated transcriptional changes across multiple RNA molecules simultaneously [12,32]. Targeted RT-qPCR assays are particularly relevant for forensic applications because they require relatively small quantities of biological material and can be applied to selected panels of candidate RNA markers.

However, RNA analysis presents several analytical challenges in forensic contexts. Unlike DNA, RNA molecules are inherently more susceptible to degradation due to enzymatic activity and environmental exposure. Consequently, RNA quality can be strongly affected by postmortem interval, storage conditions, temperature, and microbial contamination. For this reason, forensic RNA studies often incorporate RNA integrity assessment metrics, such as the RNA Integrity Number (RIN), to evaluate sample quality prior to downstream analysis and to minimise analytical artefacts.

Another important consideration in RNA-based age estimation is tissue specificity. RNA expression patterns vary considerably between cell types because transcription reflects dynamic biological processes occurring within specific tissues. As a result, prediction models developed using one tissue type, such as peripheral blood, may not perform reliably when applied to other biological materials encountered in forensic investigations.

Environmental and physiological factors can also influence RNA expression patterns. Exposure to ultraviolet radiation, oxidative stress, inflammation, or disease processes has been shown to modulate miRNA and lncRNA expression, potentially affecting the accuracy of age prediction models [34,35,36]. These influences may introduce variability in RNA-based age estimates, particularly when samples originate from individuals with differing health status or environmental exposures.

Although RNA-based biomarkers offer promising insights into molecular aging processes, several limitations currently restrict their forensic implementation. RNA degradation, variability in expression patterns across tissues, and sensitivity to environmental influences can all affect the reliability of transcription-based age prediction models. Furthermore, many RNA biomarker panels have been evaluated primarily in controlled laboratory studies using relatively homogeneous sample populations.

Consequently, RNA-based approaches remain less extensively validated than DNA methylation-based methods, which currently demonstrate more consistent predictive performance across multiple forensic sample types. Additional research is needed to establish reproducible RNA marker panels, develop robust analytical workflows for degraded forensic material, and evaluate model performance across diverse populations and forensic scenarios. At present, RNA-based biomarkers should be considered promising but largely experimental tools for age estimation, with further methodological standardisation and validation required before routine forensic implementation can be achieved.

## 8. Mitochondrial DNA-Based Techniques

Mitochondrial DNA (mtDNA) has long played an important role in forensic genetics due to several unique biological characteristics. Each cell contains hundreds to thousands of copies of the mitochondrial genome, making mtDNA more readily detectable in highly degraded biological material than nuclear DNA. For this reason, mtDNA analysis is widely used in forensic investigations involving burned remains, aged skeletal material, hair shafts, and other compromised biological samples [13,37,38]. In forensic practice, mtDNA has traditionally been employed for individual identification and maternal lineage analysis, particularly when nuclear DNA profiles cannot be obtained.

It is important to distinguish this well-established forensic use of mtDNA from its more recent investigation as a potential biomarker for age estimation. While mtDNA plays a critical role in cellular metabolism and aging biology, its application for chronological age prediction remains considerably less developed than its use in forensic identification.

Age-related changes in mtDNA have been attributed primarily to the accumulation of mutations, deletions, and alterations in heteroplasmy levels. Heteroplasmy refers to the coexistence of multiple mitochondrial genome variants within a single individual or cell population. Because mitochondria are exposed to high levels of reactive oxygen species generated during cellular respiration, the mitochondrial genome may accumulate mutations over time, reflecting cumulative oxidative damage associated with aging [39].

Several studies have suggested that the frequency of mtDNA mutations and the degree of heteroplasmy may increase with chronological age. Advances in next-generation sequencing technologies have enabled the detection of low-frequency heteroplasmic variants, allowing researchers to explore whether mitochondrial mutation profiles could serve as components of molecular aging markers [39,40]. In theory, the progressive accumulation of mitochondrial mutations could provide information about biological aging processes.

Despite these theoretical advantages, the use of mtDNA as a standalone biomarker for age estimation faces several important limitations. Mutation accumulation in mitochondrial genomes shows substantial inter-individual variability, influenced by factors such as genetic background, metabolic activity, environmental exposure, and disease status [40]. In addition, heteroplasmy patterns vary considerably between tissues, meaning that mitochondrial mutation profiles observed in blood samples may differ from those found in bone, muscle, or other biological materials.

Another practical challenge relates to contamination risk and technical variability in mtDNA sequencing. Because mitochondrial genomes are highly abundant and easily amplified, even small amounts of contaminating DNA can affect analytical results. Careful laboratory procedures and sequencing controls are therefore required to ensure reliable interpretation of mitochondrial data [38].

Although mitochondrial DNA plays an essential role in forensic identification, its use in chronological age estimation remains exploratory. Current evidence suggests that mtDNA mutation patterns alone do not provide predictive accuracy comparable to that achieved with DNA methylation-based age estimation models. Nevertheless, mitochondrial biomarkers may still provide complementary information when integrated with other molecular aging indicators.

Future research may explore the integration of mitochondrial mutation data with epigenetic, transcriptomic, or proteomic biomarkers to improve multi-marker age prediction models. However, at present, mitochondrial DNA should be considered an emerging research area rather than a validated forensic age estimation method.

## 9. Proteomic Approaches

Proteomic analysis has emerged as a complementary molecular strategy for investigating age-related biological changes at the level of protein expression and post-translational modification. Because proteins represent the functional products of gene expression, age-associated alterations in protein abundance, structure, and modification patterns may reflect physiological processes associated with aging. In forensic contexts, proteomic biomarkers have been explored in biological materials such as bone, teeth, and blood-derived samples, where age-dependent protein changes may be preserved over extended periods [41].

Advances in mass spectrometry (MS)-based proteomics, particularly liquid chromatography–tandem mass spectrometry (LC–MS/MS), have enabled the identification and quantification of complex protein mixtures with high analytical sensitivity. These techniques allow researchers to detect age-associated differences in protein expression levels, peptide fragmentation patterns, and post-translational modifications across multiple proteins simultaneously [41]. In mineralised tissues such as bone and teeth, certain structural proteins and extracellular matrix components may persist for long periods, making them attractive targets for forensic biomarker discovery.

A substantial portion of forensic proteomics research has focused not on chronological age estimation, but rather on postmortem interval (PMI) estimation and protein degradation processes, sometimes referred to as thanatochronology. In these studies, investigators examine how protein degradation patterns change after death in order to estimate the time since death. Protein breakdown rates can be influenced by environmental factors such as temperature, microbial activity, humidity, and burial conditions, making proteomics a valuable tool for PMI estimation in forensic investigations [42].

Although these studies provide important insights into protein stability and degradation dynamics, their primary objective differs from that of chronological age estimation. As a result, it is important to distinguish between proteomic biomarkers associated with postmortem processes and those that may correlate with the biological aging of individuals prior to death.

Several factors complicate the use of proteomic biomarkers for forensic age estimation. Protein stability can be strongly influenced by environmental conditions, sample preservation, and postmortem degradation, which may alter protein expression patterns independently of chronological age. In addition, protein extraction and analysis often require specialised instrumentation and technical expertise, including high-resolution mass spectrometry platforms and complex bioinformatic workflows [42].

Another limitation is the relative scarcity of large validation studies examining age-associated proteomic markers across diverse populations and tissue types. Many studies remain exploratory, focusing on biomarker discovery rather than the development of validated prediction models suitable for forensic casework.

Despite these challenges, proteomic approaches may still contribute valuable information to molecular age estimation frameworks. In particular, mineralised tissues such as bone and teeth, which can preserve proteins over extended periods, represent promising targets for age-related biomarker analysis. Combining proteomic data with other molecular indicators, such as DNA methylation or transcriptomic markers, may improve predictive accuracy in multi-omic age estimation models.

However, at present, proteomic age estimation remains an emerging research area with limited standardised methodologies for routine forensic implementation. Further research is needed to identify reproducible protein biomarkers, develop validated prediction models, and evaluate their performance in realistic forensic scenarios involving degraded samples.

## 10. Telomere Length Analysis

Telomeres are repetitive nucleotide sequences located at the ends of chromosomes that protect chromosomal DNA from degradation and inappropriate repair. During normal cellular replication, telomeres progressively shorten due to the end-replication problem and cumulative oxidative stress, processes that contribute to cellular senescence and organismal aging [43,44,45,46]. Because telomere shortening generally occurs over the lifespan of an individual, telomere length has been investigated as a potential biomarker for estimating biological or chronological age.

Several laboratory techniques have been developed to measure telomere length. One of the most widely used approaches is quantitative polymerase chain reaction (qPCR), which estimates relative telomere length by comparing telomeric repeat copy number with that of a single-copy reference gene [47]. This method is attractive for forensic research because it requires relatively small amounts of DNA and can be applied to degraded samples.

Another commonly used technique is telomere restriction fragment (TRF) analysis, which measures telomere length through Southern blotting of digested genomic DNA [48]. Although TRF analysis provides a more direct assessment of telomere length distribution, it requires higher DNA input and more extensive laboratory processing. More recently, sequencing-based approaches have also been explored to improve resolution and allow more detailed analysis of telomeric regions [48].

Despite the biological relationship between telomere shortening and aging, the use of telomere length for forensic age estimation faces several significant limitations. Telomere length exhibits substantial inter-individual variability, influenced by genetic background, lifestyle factors, stress, disease status, and environmental exposures [48]. Consequently, individuals of the same chronological age may show considerable differences in telomere length.

In addition, the relationship between telomere length and chronological age is not linear across the lifespan. Telomere shortening occurs rapidly during early development and childhood but tends to stabilise during adulthood, reducing the predictive value of telomere measurements in older individuals [49]. Telomere length also varies between tissues, meaning that measurements obtained from blood samples may not accurately represent telomere dynamics in other biological materials encountered in forensic investigations.

Because of these limitations, telomere length is generally considered insufficient as a standalone biomarker for chronological age estimation. Several studies have reported relatively large prediction errors when telomere length is used alone, particularly in adult populations [50,51,52,53,54]. However, telomere measurements may still provide complementary information about biological aging when combined with other molecular markers.

Integrative approaches that combine telomere length with DNA methylation, RNA expression, or proteomic biomarkers may improve the overall accuracy of molecular age prediction models by capturing multiple biological dimensions of aging [55]. Nevertheless, substantial methodological standardisation and large-scale validation studies would be required before telomere-based age estimation could be considered for routine forensic application.

## 11. Discussion

**Figure 1:**
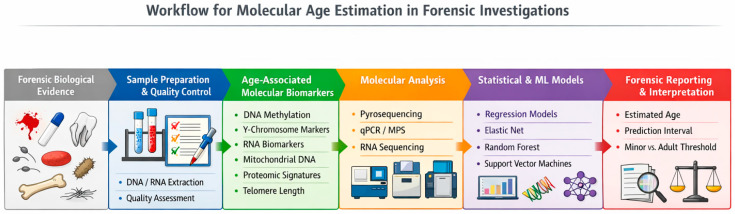
Workflow for molecular age estimation in forensic investigations. Schematic representation of the analytical workflow used in molecular approaches to forensic age estimation. Biological evidence collected from forensic samples (e.g., blood, saliva, semen, bone, or teeth) undergoes DNA or RNA extraction followed by quality assessment. Age-associated molecular biomarkers, including DNA methylation markers, Y-chromosome markers, RNA expression biomarkers, mitochondrial DNA variants, proteomic signatures, and telomere length, are analysed using appropriate molecular platforms such as pyrosequencing, quantitative PCR, massively parallel sequencing, RNA sequencing, or mass spectrometry. Resulting molecular data are processed using statistical or machine learning models to estimate chronological age. Final interpretation includes reporting prediction intervals and uncertainty to support forensic decision-making.

Forensic age estimation (Figure 1) has undergone a significant conceptual transition over the past two decades, evolving from predominantly morphology-based approaches toward increasingly sophisticated molecular and computational frameworks. Traditional anthropological and radiological methods remain foundational within forensic practice; however, their limitations, including subjectivity, population dependency, and broad age ranges, have driven the search for more objective and reproducible alternatives [4,20]. Molecular techniques offer a promising pathway toward improving accuracy, particularly in scenarios involving minimal or degraded biological material, where conventional skeletal assessment may be impractical or impossible.

Among the molecular strategies discussed, DNA methylation currently represents the most mature and widely validated approach to forensic age estimation. Epigenetic clocks have demonstrated relatively low prediction error compared with other biomarkers, highlighting their potential for practical forensic application [10,56]. Nevertheless, no single molecular marker fully captures the complexity of human aging. Biological aging reflects the cumulative interaction of genetic regulation, environmental exposure, metabolic processes, and cellular stress, suggesting that integrative multi-marker models may provide the most reliable predictions. Combining epigenetic, transcriptomic, proteomic, mitochondrial, and telomere-based data has emerged as a key research direction, enabling the development of multi-omic frameworks that account for multiple dimensions of biological aging simultaneously.

Advances in computational modelling have played an increasingly important role in the development of molecular age estimation methods. Many biomarker-based approaches generate large datasets that include multiple molecular variables, such as DNA methylation levels across numerous CpG sites, RNA expression profiles, or proteomic measurements. Machine learning algorithms provide analytical frameworks capable of identifying complex relationships between these variables and chronological age, enabling the development of predictive models that extend beyond traditional linear regression approaches [15,57].

In the context of DNA methylation-based age estimation, machine learning methods have been widely used to construct epigenetic clocks that integrate information from multiple CpG loci to generate age predictions. Techniques such as elastic net regression, random forest models, and support vector machines have been applied to identify informative biomarker combinations and optimize prediction accuracy. These computational approaches can improve model performance by capturing non-linear relationships between molecular markers and chronological age, particularly when large multi-omic datasets are available [57].

Machine learning techniques are also increasingly explored for multi-omic age prediction models that combine different classes of biomarkers, including methylation markers, RNA expression profiles, proteomic signatures, and mitochondrial mutation patterns. Integrating multiple biological layers may improve the robustness of age predictions by capturing complementary aspects of the aging process. However, the application of machine learning in forensic age estimation also introduces important considerations related to model transparency, reproducibility, and interpretability, which are essential for legal and forensic contexts.

Although computational approaches have shown considerable promise in improving age prediction models, many existing algorithms have been developed using relatively homogeneous research cohorts and controlled laboratory conditions. Additional research is therefore needed to evaluate the performance of machine learning-based age estimation models across diverse populations, degraded forensic samples, and realistic casework scenarios. Establishing standardized analytical pipelines and transparent reporting of prediction uncertainty will be critical for translating these computational approaches into operational forensic practice.

Despite these advances, several challenges must be addressed before molecular age estimation can be routinely implemented in forensic casework. Standardization represents one of the most significant barriers. Differences in laboratory protocols, sequencing platforms, analytical pipelines, and statistical models can lead to variability in age prediction outcomes, raising concerns regarding reproducibility and legal admissibility. Establishing consensus guidelines for biomarker selection, assay validation, and data interpretation will be essential to ensure consistent and defensible forensic practice. Additionally, many molecular techniques require specialized infrastructure and technical expertise that may not be accessible to all forensic laboratories, highlighting the need for cost-effective and scalable analytical strategies.

Ethical considerations also play a crucial role in shaping the future of molecular age estimation. While traditional radiological methods raise concerns regarding radiation exposure and consent [21], molecular techniques introduce new questions related to genetic privacy and the potential inference of sensitive biological information beyond age alone. For example, epigenetic or transcriptomic data may inadvertently reveal health status or environmental exposures, necessitating careful consideration of ethical guidelines and data governance policies within forensic frameworks.

Another key area for future research involves improving population diversity within age estimation datasets. Many existing biomarker panels have been developed using relatively homogeneous cohorts, which may limit generalizability across global populations. Expanding validation studies to include diverse demographic groups will be critical to minimize bias and ensure equitable forensic application. Longitudinal studies examining biomarker stability across the lifespan may further enhance our understanding of biological aging trajectories and refine predictive accuracy.

Looking forward, the integration of molecular and traditional approaches may provide the most effective strategy for advancing forensic age estimation. Rather than replacing anthropological methods, molecular biomarkers should be viewed as complementary tools capable of enhancing confidence in age predictions when used alongside established forensic practices. The advantages and limitation of each tool as highlighted in (Table 1) provides for the development of hybrid workflows, combining skeletal assessment, epigenetic profiling, and computational modelling, could enable more precise and scientifically grounded estimations while maintaining transparency and interpretability.

Therefore, the field of forensic age estimation is entering a transformative phase driven by advances in molecular biology and data science. Continued interdisciplinary collaboration will be essential to refine biomarker panels, establish standardized methodologies, and ensure responsible integration into forensic practice. As research progresses, molecular age estimation has the potential to move beyond experimental application toward routine forensic implementation, offering improved accuracy, reproducibility, and investigative value while maintaining ethical and legal integrity.

## 12. Conclusions

Molecular approaches to forensic age estimation have advanced substantially over the past two decades, providing new opportunities to complement traditional anthropological and radiological methods. Among the molecular strategies investigated to date, DNA methylation-based models currently demonstrate the strongest predictive performance and represent the most mature approach for forensic age estimation, with several targeted marker panels achieving mean absolute errors of approximately three to six years in specific tissues. These epigenetic approaches have shown promising results across commonly encountered forensic sample types, including blood, saliva, and buccal cells, and continue to benefit from advances in sequencing technologies and statistical modelling.

In contrast, other molecular biomarkers, such as Y-chromosomal markers, RNA expression profiles, mitochondrial DNA variation, proteomic signatures, and telomere length, remain at earlier stages of research development. While these biomarkers provide valuable insights into biological aging processes, their predictive accuracy, reproducibility, and validation across forensic sample types are currently more limited. Factors such as tissue specificity, environmental exposure, disease status, and sample degradation may introduce variability that complicates the interpretation of age-related molecular signals.

Before molecular age estimation can be widely implemented in forensic casework, several critical challenges must be addressed. These include the need for standardized analytical protocols, external validation across independent populations, robust performance evaluation using degraded forensic samples, and transparent statistical modelling approaches that provide clear prediction intervals and uncertainty estimates. In addition, forensic laboratories must ensure that molecular age estimation methods are compatible with legal standards of scientific reliability and admissibility.

Future developments are likely to focus on improving targeted DNA methylation panels, integrating multiple biomarker classes through multi-omic modelling approaches, and refining computational methods capable of analyzing complex biological datasets. With continued methodological refinement and rigorous validation, molecular age estimation techniques have the potential to become valuable complementary tools within the broader framework of forensic identification and human biological profiling.

## Figures and Tables

**Table 1:** Comparative overview of molecular approaches for forensic age estimation. Summary of major molecular strategies currently investigated for forensic age prediction, including DNA methylation, Y-chromosome markers, RNA-based biomarkers, mitochondrial DNA analysis, proteomic approaches, and telomere length assessment. The table summarizes typical sample types, analytical platforms, reported prediction errors (e.g., MAE), validation status, key limitations, and current level of forensic readiness. DNA methylation-based models currently show the most consistent predictive accuracy and validation across forensic sample types, whereas other biomarker classes remain largely exploratory.

Biomarker Type	Typical Sample Types	Analytical Platform	Typical Prediction Error (MAE)	Validation Level	Major Limitations	Forensic Readiness
DNA Methylation (Epigenetic markers)	Blood, saliva, buccal cells, semen	Pyrosequencing, qPCR, Massively Parallel Sequencing (MPS), Illumina methylation arrays	~3–6 years depending on marker panel and tissue	Multiple cohort studies; several forensic-focused validation studies	Tissue specificity, environmental influences, population variability	Most advanced; promising for forensic implementation
Y-Chromosome Methylation Markers	Blood, semen (male-specific samples)	Targeted methylation assays, sequencing	Limited data; generally higher error than autosomal methylation models	Early-stage research studies	Applicable only to males; limited number of informative loci	Supplementary research tool
mLOY (Mosaic Loss of Y Chromosome)	Blood (male samples)	Genome sequencing, cytogenetic detection	Not well established for age prediction	Exploratory studies	Strongly influenced by smoking, disease, inflammation	Experimental biomarker
RNA Expression Biomarkers (miRNA, lncRNA, circRNA)	Blood, tissues	RT-qPCR, RNA sequencing, microarrays	Variable; not consistently validated	Early-stage biomarker discovery studies	RNA degradation, tissue specificity, environmental influences	Experimental; requires further validation
Mitochondrial DNA Mutations/Heteroplasmy	Degraded tissues, bone, hair shafts	Next-generation sequencing	Predictive accuracy not well established	Limited exploratory studies	High variability between individuals and tissues	Exploratory research area
Proteomic Biomarkers	Bone, teeth, blood	LC–MS/MS mass spectrometry	Limited validated prediction models	Small exploratory studies	Environmental degradation, technical complexity	Emerging research area
Telomere Length	Blood, tissues	qPCR, Southern blot (TRF), sequencing	Large prediction error; weak correlation in adults	Numerous aging studies but limited forensic validation	High inter-individual variability; tissue differences	Limited utility as standalone marker

## Data Availability

No new data were created or analyzed in this study. Data sharing is not applicable to this article.
